# Early Morphological Changes of the Rectus Femoris Muscle and Deep Fascia in Ullrich Congenital Muscular Dystrophy

**DOI:** 10.3390/ijerph19031252

**Published:** 2022-01-23

**Authors:** Patrizia Sabatelli, Luciano Merlini, Alberto Di Martino, Vittoria Cenni, Cesare Faldini

**Affiliations:** 1 Unit of Bologna, CNR-Institute of Molecular Genetics “Luigi Luca Cavalli-Sforza”, 40136 Bologna, Italy; vittoria.cenni@cnr.it; 2 IRCCS Istituto Ortopedico Rizzoli, 40136 Bologna, Italy; 3 Department of Biomedical and Neuromotor Sciences, University of Bologna, 40126 Bologna, Italy; luciano.merlini@unibo.it (L.M.); or dimartino.cbm@gmail.com (A.D.M.); cesare.faldini@ior.it (C.F.); 4 Clinica Ortopedica e Traumatologica I, IRCCS Istituto Ortopedico Rizzoli, 40136 Bologna, Italy; 5 Sidney Kimmel Medical College, Thomas Jefferson University, Philadelphia, PA 19107, USA

**Keywords:** collagen VI, Ullrich congenital muscular dystrophy, fatty infiltration, muscle fiber atrophy, telocytes

## Abstract

Ullrich congenital muscular dystrophy (UCMD) is a severe form of muscular dystrophy caused by the loss of function of collagen VI, a critical component of the muscle-tendon matrix. Magnetic resonance imaging of UCMD patients’ muscles shows a peculiar rim of abnormal signal at the periphery of each muscle, and a relative sparing of the internal part. The mechanism/s involved in the early fat substitution of muscle fiber at the periphery of muscles remain elusive. We studied a muscle biopsy of the rectus femoris/deep fascia (DF) of a 3-year-old UCMD patient, with a homozygous mutation in the *COL6A2* gene. By immunohistochemical and ultrastructural analysis, we found a marked fatty infiltration at the interface of the muscle with the epimysium/DF and an atrophic phenotype, primarily in fast-twitch fibers, which has never been reported before. An unexpected finding was the widespread increase of interstitial cells with long cytoplasmic processes, consistent with the telocyte phenotype. Our study documents for the first time in a muscle biopsy the peculiar pattern of outside-in muscle degeneration followed by fat substitution as already shown by muscle imaging, and an increase of telocytes in the interstitium of the deep fascia, which highlights a potential involvement of this structure in the pathogenesis of UCMD.

## 1. Introduction

Collagen VI is a microfibrillar collagen expressed in the matrix of most tissues. The best-characterized and most widely expressed form of collagen VI is the [α1, α2, α3] heterotrimer [1]. The α5 and α6 chains are homologues of the α3 chain, but display a more restricted and differential distribution pattern [2,3]. Ultrastructurally, collagen VI forms a network of beaded filaments anchored to the cell surface [4]. By interacting with cell membrane receptors and extracellular components, collagen VI fibrils form a network that connects the cell cytoskeleton to the extracellular environment [1,5]. The role of collagen VI is particularly relevant for muscle-tendon unit function. The muscle-tendon unit involves the connection between the muscles and tendons, through which contractile forces are generated and transmitted [6]. The muscular/deep fascia (DF), the connective tissue sheath surrounding skeletal muscle, contributes to the transmission of muscular force between adjacent synergistic muscles [7,8]. A detailed morphological study of the DF has been reported in adult subjects [9], but a description of the DF in children is still lacking. In skeletal muscle, collagen VI localizes in the basement of muscle fibers and blood vessels and in the interstitium (epimysium, perimysium, and endomysium), anchoring the interstitial matrix. In tendons, in addition to interfibrillar distribution, collagen VI is enriched in the pericellular matrix of tenocytes, where it regulates critical cellular functions by interacting with cell membrane receptors and extracellular matrix proteins [10]. Although poorly documented, collagen VI has been observed in the muscular fascia [11], likely contributing to the general role of the extracellular matrix (ECM) in providing resistance to tension and stretch, which commonly occurs in the fascial system. In addition to its mechanical/structural role, collagen VI regulates a variety of cellular functions including proliferation, migration, differentiation, autophagy, and stemness maintenance [1].

Mutations in genes encoding the α1, α2, and α3 chains of collagen VI cause several forms of muscular dystrophy, namely, Ullrich congenital muscular dystrophy (UCMD [MIM 254090]), Bethlem myopathy (BM [MIM 158810]), and myosclerosis myopathy (MM [MIM 255600]) [12]. UCMD represents the most severe form. It is characterized by congenital muscle weakness with axial and proximal joint contractures and coexisting distal joint hypermobility [12]. The presentation is usually at birth, with hypotonia, congenital hip dislocation, prominent calcanei, and a transient kyphotic deformity. Motor milestones are delayed, and most children never acquire the ability to walk independently [12]. Follicular hyperkeratosis over the extensor surfaces of upper and lower limbs, keloids, and cigarette paper scar formation are common. BM and MM display a distinct and less-severe phenotype [12]. Prevalence is estimated at 0.77 in 100,000 for BM and 0.13 in 100,000 for UCMD [13].

Muscle biopsies from UCMD patients show a complete or partial reduction of collagen VI [14], while in BM patients the protein pattern may be normal [15] or moderately reduced [16], depending on the mode of inheritance of mutation (dominant vs. recessive, respectively). In UCMD patients with partial deficiency, there is a specific collagen VI reduction in the basement membrane of muscle fibers and blood vessels. The absence of collagen VI in the basement membrane affects the anchorage of muscle fibers to the interstitium, as demonstrated by the electron microscope observation of an empty space between the basal lamina and the endomysial matrix [17]. Collagen VI is mainly produced by fibroblasts resident in the tissue [18]. Cultured skin fibroblasts are usually helpful for studies of collagen VI mRNA and protein synthesis, instead, the analysis of skin biopsies is not always informative in patients with partial deficiency, as collagen VI may be expressed at normal levels [19].

A distinctive feature of collagen VI-related myopathies is provided by muscle imaging. In patients with BM, we have observed by muscle computed tomography (CT) that the rectus femoris and vastus lateralis in particular present early fat substitution at their periphery, with a long sparing of the central part [20], and that the same pattern was also present in UCMD [15]. This peculiar pattern of outside–in muscle degeneration followed by fat substitution has been subsequently confirmed with different muscle imaging techniques [21,22,23,24,25,26]. These alterations represent a unique feature of collagen VI-related myopathies and are predictive of mutations in collagen VI genes.

Regarding the pathogenesis of collagen VI-related myopathies, critical events are mitochondrial dysfunction due to inappropriate opening of the mitochondrial permeability transition pore [27] and inadequate removal of altered mitochondria by defective autophagy [28], as demonstrated in collagen VI-deficient mice [27,28], zebrafish [29], and in UCMD and BM patients [30,31].

Assuming that the defect of collagen VI is present throughout the muscle, it remains to be explained why the external fibers, in contact with the DF, rapidly degenerate while internal ones are protected from its damaging effect. This may suggest that fascia, mechanical stress and/or increase in metabolic demand stress are directly responsible for the degenerative process, possibly opening new therapeutic or conservative treatment options.

To our knowledge, a study of early changes occurring at the muscle perifascicular area as detected by CT [15,20], magnetic resonance imaging (MRI) [32], and ultrasound (US) [21], has never been reported. We studied the rectus femoris/DF biopsy of a 3-year-old UCMD patient. We found fatty proliferation at the interface of the muscle with the epimysium/DF, an atrophic phenotype primarily in fast-twitch fibers and proliferation of telocytes, changes never before reported in UCMD muscle biopsies.

## 2. Materials and Methods

*Biopsies.* We obtained muscle biopsies of the rectus femoris/DF in a 3-year-old child with UCMD with an homozygous mutation in *COL6A2* (c.2572C > T) [33], in a 4-year-old non-dystrophic patient undergoing surgical treatment for open fracture of the femur, and in a 2-year-old patient with Duchenne muscular dystrophy (DMD) with a nonsense mutation of exon 70 (c.10108C > T [p.R3370X]). The UCMD patient and the non-dystrophic control both had a normal creatine kinase level, while the disease control had a very high CK, as expected in Duchenne muscular dystrophy. Informed consent for participation in the study was obtained from the parents of the children. The study was conducted in accordance with the Declaration of Helsinki, and the protocol was approved by the Ethics Committee of the Rizzoli Orthopedic Institute (CE 0007151).

*Immunofluorescence study*. Seven-micron-thick frozen sections were incubated with primary anti-collagen VI (Millipore, Burlington, MA, USA), anti-collagen I (Abcam, Cambridge, UK), anti-perilipin (Santa Cruz, Dallas, TX, USA), anti-developmental, anti-neonatal, fast (Leica, Wetzlar, Germany) and slow myosin heavy chain (Santa Cruz, Dallas, TX, USA), anti-CD34 (Biolegend, San Diego, CA, USA), and anti-nidogen (Millipore, Burlington, MA, USA) antibodies and incubated with FITC, TRITC, or CY5-conjugated anti-mouse or anti-rabbit secondary antibodies (DAKO, Glostrup, Denmark). FITC-conjugated phalloidin (Sigma, St. Louis, MO, USA) was used for the filamentous actin staining of myofibers. Cell nuclei were stained with 1 µg/mL DAPI (Sigma-Aldrich, St. Louis, MO, USA). Samples were mounted with an anti-fading reagent (Molecular Probes, Eugene, OR, USA) and observed with a Nikon epifluorescence microscope.

*Transmission electron microscopy.* Rectus femoris muscle fragments were fixed with 2.5% glutaraldehyde in 0.1 M cacodylate buffer and 1% osmium tetroxide and embedded in Epon812 epoxy resin, following standard procedures. Semithin sections were stained with toluidine blue. Ultrathin sections were counterstained with uranyl acetate and lead citrate and observed with a JEOL JEM-1011 transmission electron microscope operated at 100 kV.

## 3. Results

**Figure 1:**
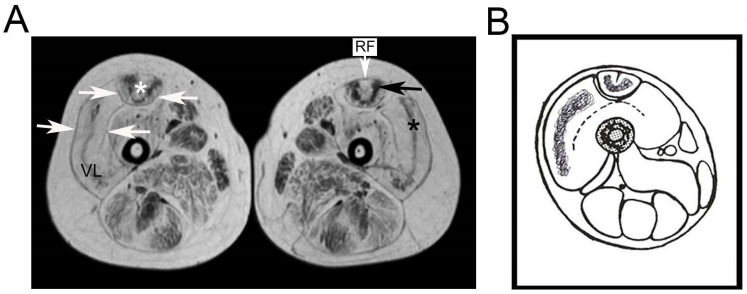
Imaging of thigh muscles in the UCMD patient. (**A**) Muscle magnetic resonance of the thigh in the UCMD patient at the age of 6 years. The vastus lateralis (VL) and rectus femoris (RF) show a proliferation of fat tissue as indicated by the area of high signal density closely associated with the peripheral fascia (white arrows). In addition, the rectus femoris shows the typical area of high signal surrounding the central fascia (“central shadow sign”—white asterisk). A central part of relative muscle substitution sparing is present in the vastus lateralis (“sandwich sign”—black asterisk) and in the rectus femoris with a “U” pattern (black arrow). (**B**) MRI-based schematic representation of thigh muscles in the UCMD patient.

Muscle magnetic resonance of the thigh in the UCMD patient at the age of 6 years showed a rim of fat tissue in the vastus lateralis and rectus femoris, as indicated by an area of high signal density closely associated to the peripheral fascia. In addition, the rectus femoris showed the typical high-signal area surrounding the central fascia (“central shadow sign”). A central part of relative muscle substitution sparing was present in the vastus lateralis (“sandwich sign”) and in the rectus femoris with a “U” pattern (Figure 1A,B).

**Figure 2:**
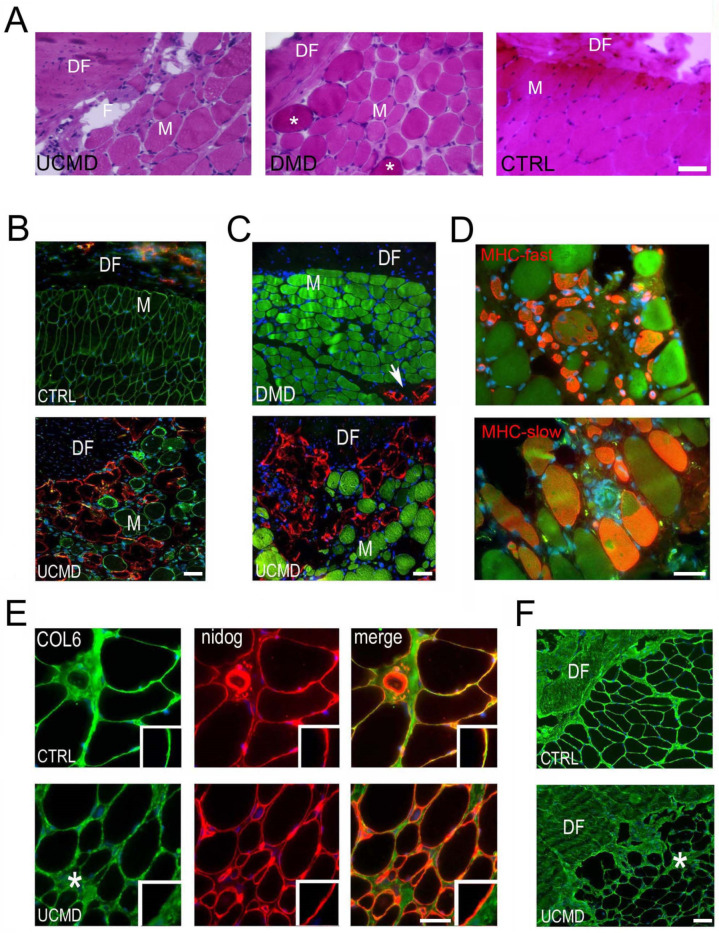
Morphological changes in the UCMD muscle/DF biopsy. (**A**) Hematoxylin and eosin staining on cross sections of the muscle/DF biopsy from the UCMD patient (UCMD), and a patient affected by Duchenne muscular dystrophy (DMD). Note the presence of an empty space (F) between the DF and the muscle fibers (M) in the UCMD biopsy, suggestive of fat tissue proliferation. Fatty infiltration is not evident at the muscle/DF interface in both DMD and non-dystrophic control (CTRL) biopsy; DMD biopsy displays the characteristic hypercontracted fibers (asterisk). Scale bar, 50 µm. (**B**) Immunofluorescence analysis of dystrophin (green) and perilipin (red) on muscle sections of the non-dystrophic control (CTRL, upper panel) and the UCMD patient (UCMD, lower panel). Fatty infiltrate, as detected by perilipin, is prominent at the muscle (M)/DF interface in the UCMD biopsy, while it is absent in the analogue region of the non-dystrophic control. Nuclear staining, DAPI. DF, deep fascia. Scale bar, 50 µm. (**C**) Anti-perilipin (red) immunostaining on DMD and UCMD muscle sections. Note the differential distribution of adipose cells (perilipin-positive) which appears mainly subfascial in UCMD, and perimysial in the DMD patient (arrow). Muscle fibers are marked with FITC-conjugated phalloidin (green). Nuclear staining, DAPI. Scale bar, 50 µm. (**D**) Fast (MHC-fast) and slow (MHC-slow) myosin heavy chain staining in UCMD muscle biopsy showing the atrophic phenotype primarily in fast-twitch fibers. Muscle fibers are labeled with FITC-conjugated phalloidin (green). Nuclear staining, DAPI. Scale bar, 50 µm. (**E**) Immunofluorescence analysis of collagen VI (COL6, green) and the basement membrane component nidogen (nidog, red), and superimposed images (merge) on non-dystrophic control (CTRL) and UCMD patient (UCMD). Note the presence of a continuous signal at the basement membrane of myofibers of the non-dystrophic control with anti-collagen VI antibody, perfectly merging with nidogen staining (orange signal in merge image). UCMD muscle displays an accumulation of collagen VI in the interstitium (asterisk) and a marked reduction in the basement membrane of muscle fibers, as indicated by the red signal in the merge image. Inserts show a detail of the muscle fiber basement membrane of the respective images. Nuclear staining, DAPI. Scale bar, 30 µm. (**F**) Low magnification of collagen VI immunolabeling on cross sections of muscle/DF biopsies showing an undulating pattern in the DF in the UCMD patient (UCMD), as compared with the diffuse and more intense pattern in the non-dystrophic control. Nuclear staining, DAPI. Scale bar, 50 µm.

In the UCMD patient, a diagnostic biopsy was obtained from the rectus femoris/DF when he was 3 years old. The morphological analysis showed a dystrophic pattern, characterized by fiber size variability, internal nuclei, increased endomysial and perimysial fibrosis, and a marked fatty infiltration at the interface of muscle with the epimysium/DF (Figure 2A). The DF displayed the typical fibrous organization characterized by undulated collagen fibers (Figure 2A), as also confirmed by immunofluorescence analysis with an antibody against anti-collagen I, a main component of the DF matrix (Figure S1A).

To better characterize the peculiar fatty infiltration at the muscle/DF interface, we checked for the expression of perilipin, an adipocyte-specific lipid-associated protein (Figure 2B,C). The immunofluorescence analysis confirmed the marked fatty infiltration at the muscle/DF interface (Figure 2B,C). By contrast, adipose cells were absent at the interface of rectus femoris/DF biopsies from a non-dystrophic control (Figure 2B) and a DMD patient (Figure 2C).

The study of developmental (Figure S1B) and neonatal (Figure S1C) myosin heavy chains in the UCMD biopsy showed the presence of small immature fibers; however, only rare fibers expressed the developmental isoform. Fast and slow myosin isoform immunohistochemistry excluded the presence of fiber type grouping and revealed the atrophic phenotype, primarily in fast-twitch fibers (Figure 2D).

The expression and distribution of collagen VI was studied by double labeling with an anti-nidogen antibody, a component of the basement membrane of muscle fibers and blood vessels. In the UCMD biopsy, collagen VI was reduced in the basement membrane of muscle fibers and accumulated in the interstitium, a pattern typical of UCMD patients’ muscles with partial deficiency (Figure 2E). By contrast, a non-dystrophic control showed a continuous collagen VI signal around muscle fibers, perfectly matching with the nidogen staining (Figure 2E). In the DF of the UCMD patient, collagen VI appeared mildly reduced and displayed an undulated pattern compared with the intense and diffuse pattern of the non-dystrophic control (Figure 2F).

**Figure 3:**
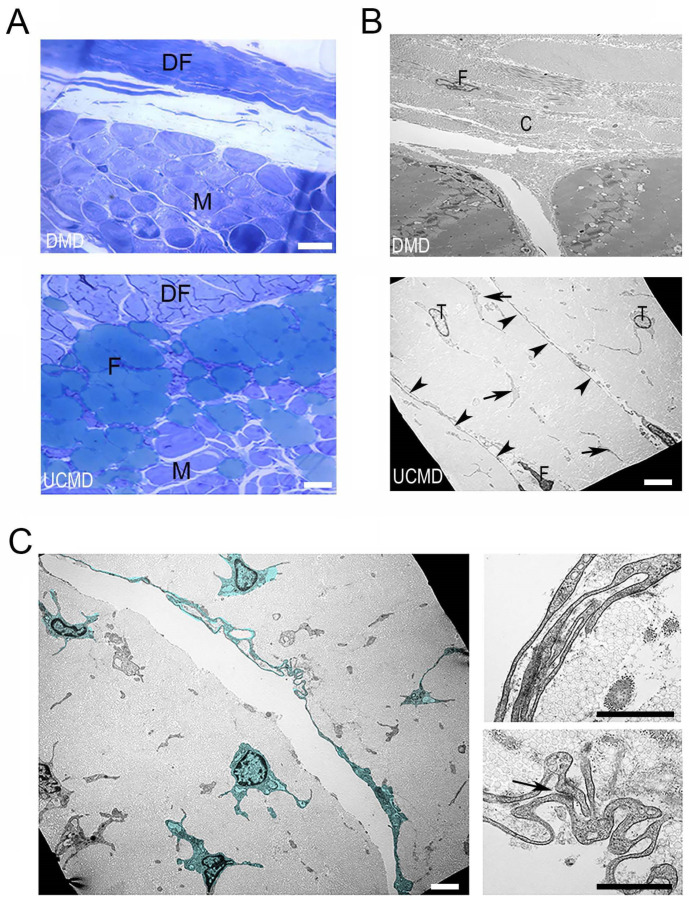
Ultrastructural changes in UCMD muscle/DF biopsy. (**A**) Semithin sections of the DMD and UCMD muscle/DF biopsies confirming the presence of ectopic fatty infiltration (F) at the interface of muscle with the epimysium/DF) in UCMD patient. On the other hand, fatty infiltration is not evident in the muscle biopsy of the DMD patient. Scale bar, 50 µm. (**B**) Transmission electron microscopy analysis of DF in DMD (DMD, upper panel) and UCMD (UCMD, lower panel) biopsies. In DMD sample, a fibroblast (F) with a typical elongated nucleus appears immersed in the DF matrix, mainly constituted by collagen fibrils (C). In addition to fibroblasts (F), the UCMD DF shows several cells with oval nuclei and long cytoplasmic processes, consistent with telocyte phenotype (T), are also present. Moniliform (arrowhead) or enlarged (arrow) telopodes are diffuse in the UCMD DF. Scale bar, 5 µm. (**C**) Digitally colored telocytes (green) showing homocellular (telocyte-to-telocyte) contacts, such as interdigitations (right, upper panel) and adherens junctions (arrow; right, lower panel). Scale bar, left panel, 10 µm, right panels, 800 nm.

**Figure 4:**
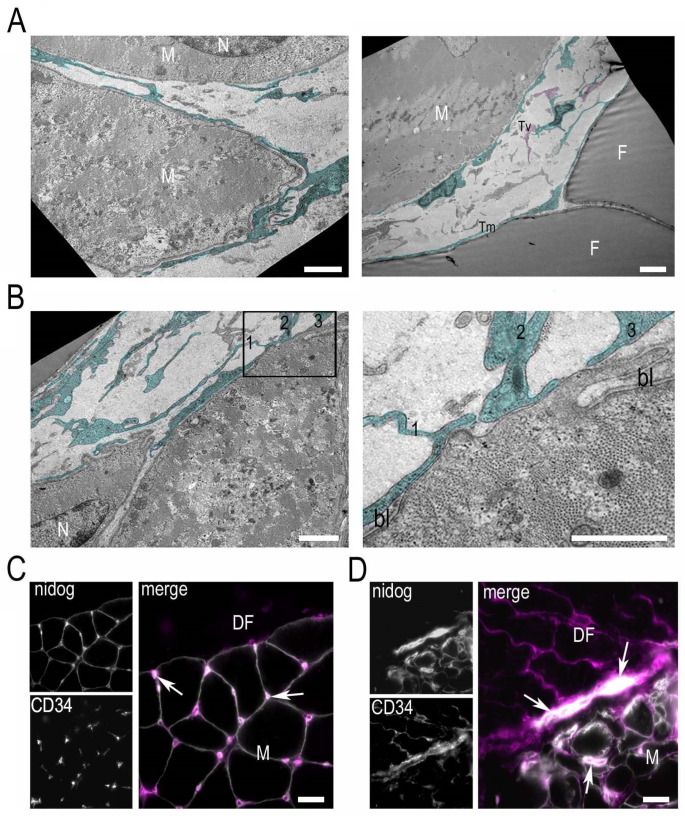
Telocyte–muscle cell relationship in the UCMD patient’s biopsy. (**A**) Ultrastructural analysis of UCMD muscle biopsy showing the presence of several telocytes (digitally colored in green) with typical moniliform (Tm) or varicose (Tv, pink colored) telopodes within the endomysium. Muscle fibers (M) and adipose cells (F) appear almost completely wrapped by telopodes. Scale bar, 1 µm. (**B**) On the left, a representative area particularly enriched in telocytes (green). In the area enclosed by the box, three telopodes (1–3) appear in close contact with a tract of sarcolemma devoid of basal lamina (enlargement in the right panel). N, nucleus; bl, basal lamina. Scale bar, 1 µm. (**C**) Immunohistochemical analysis of the basement membrane component nidogen (nidog) and CD34 (magenta in the merge panel) in the biopsy of the non-dystrophic control. Nidogen staining delineates the basement membrane of muscle fibers (M) and capillary vessels (arrows). Most of the CD34-positive cells are represented by vascular endothelial cells, as suggested by the localization in the vessel wall. Scale bar, 50 µm. (**D**) Immunohistochemical analysis of CD34 and nidogen (nidog) in the UCMD biopsy. In addition to endothelial cells (arrows), CD34 identifies several interstitial cells in the DF, where a reticular network is formed, and between muscle fibers (M). Scale bar, 50 µm.

For a better understanding of the morphological alterations, a fragment of each biopsy was processed for transmission electron microscopy analysis. Semithin cross sections of the UCMD biopsy confirmed the presence of ectopic fatty infiltration at the interface of muscle with the DF (Figure 3A). Accordingly, with the immunohistochemical analysis, adipose cells were absent in the analogue area of the DMD patient (Figure 3A). At the ultrastructural level, the DF of the UCMD patient appeared to be composed of a fibrous matrix with typically undulated collagen fibers, displaying normal size and morphology (Figure 3B). In addition to fibroblasts, which represent the main cell type in normal fascia, we noted numerous telocytes, stromal cells distinct from fibroblasts for their long moniliform cytoplasmic processes (telopodes) (Figure 3B,C); telopodes formed homocellular (telocyte-to-telocyte) contacts, featuring interdigitations and adherens junctions (Figure 3C). Telocytes were also frequently detected in the muscle interstitium, in close relationship with muscle fibers, adipocytes (Figure 4A), and blood vessels (Figure S1D). Aspects of telopodes passing through fragmented basal lamina and contacting the underlying muscle fiber membrane were frequently detected (Figure 4B). The abundance of telocytes in UCMD biopsy was also confirmed by the immunohistochemical analysis of CD34, a reliable marker for telocyte detection in situ. To discriminate CD34-positive endothelial cells from telocytes, sections were double-labeled with an anti-nidogen antibody, a component of the vessel basement membrane. In agreement with ultrastructural observations, numerous CD34-positive cells were detected in the DF and the interstitium of muscle of the UCMD patient (Figure 4D), while in the non-dystrophic control they were less diffuse and mainly associated with blood vessels (Figure 4C).

## 4. Discussion

The principal aim of our study was to shed light on the mechanism(s) leading to the loss of muscle fibers at the periphery of muscle, a pathognomonic sign of collagen VI-related myopathies.

We report that the histopathological changes occurring at the periphery of the rectus femoris muscle of a 3-year-old UCMD patient are consistent with an active dystrophic process, as indicated by the presence of fatty infiltration. This pattern works perfectly with the fat substitution of peripheral muscle fibers documented by muscle imaging in this patient with UCMD and in additional patients reported in our study [15] and other studies [21,22,23,25,26,32]. Previous studies on muscle biopsies from UCMD patients reported a variable histopathological pattern, ranging from mild myopathic [34,35] to dystrophic [36], independent of patient age [35]. It is conceivable that the observed variability may be related to the muscle or the area of biopsy sampling (peripheral vs. internal).

We found an atrophic phenotype, prevalently of fast-twitch fibers. Muscle-fiber atrophy has been described as a prevalent mechanism in biopsies obtained from patients with UCMD; however, it was mainly observed in type I fibers [34]. A bimodal distribution of type I fibers, one population of which was extremely atrophic [36], was also reported. Muscle wasting can occur through multiple distinct signaling pathways, with differential sensitivity levels between selective skeletal-muscle-fiber subtypes. Fast-twitch glycolytic fibers are more sensitive to a variety of conditions related to different signaling pathways, including autophagy [37]. It is interesting that *Col6a1*^-/-^ mice and patients display reduced autophagy flux [28]; reactivation of autophagy is protective against muscle mass loss in knock-out mice [28], while in BM and UCMD patients it improves the mitochondrial performance and the rate of apoptosis, downstream consequences of the autophagy defect [31]. On the other hand, the involvement of type II fibers as early changes in UCMD muscle could be consistent with the reported prevalence of type I fibers [36], which, moreover, are less sensitive to autophagy deficiency [37]. Slow and fast-twitch fibers display distinct mechanical behavior, metabolism, and signaling pathways [38]. Physical exercise has a detrimental effect on *Col6a1^-/-^* animals and leads to a muscle pathology closer to the human UCMD condition [38]. This is because physical exercise stimulates autophagy in muscles of wild-type mice. However, this stimulus does not trigger autophagy in collagen VI-deficient mice, and it has detrimental effects on *Col6a1^-/-^* muscles [38]. Thus, the inability to properly activate autophagy in diseased muscles under conditions of intense workload and increased need for energy sources may lead to deterioration of muscle-fiber defects.

Regeneration was evident in the muscle biopsy of our UCMD patient, as indicated by the presence of small immature fibers positive to neonatal myosin heavy chains. However, rare fibers expressed the developmental isoform, suggesting a block of the regenerative/maturation process. This observation is consistent with previous works on UCMD biopsies, which reported prominent [35,36] but abnormal [36] regeneration in UCMD muscle biopsies. Lack of collagen VI in *Col6a1^-/-^* mice causes impaired muscle regeneration and reduced satellite cell self-renewal capability after injury [39]. The mechanism underlying the defective regeneration in collagen VI-deficient mice and patients is still under investigation. However, it is interesting to note that in two pilot trials with cyclosporine A (CsA) in UCMD patients, aimed at the correction of mitochondrial dysfunction [40,41], we noted an increase of developmental myosin heavy chain-positive fibers, which are rare in patients under basal conditions. This suggests that the recovery of mitochondrial function may have a positive effect on regenerative potential. In addition, CsA treatment, by activating autophagy [28], may contribute to the recovery of a critical mechanism for muscle regeneration/differentiation [42,43].

An unexpected observation in our study was the diffuse and significant increase of cells with morphological and ultrastructural features of telocytes. Telocytes were closely associated with adipose, vessel, and muscle cells. Ultrastructurally distinguishable from other interstitial cells because very long cytoplasmic processes (telopodes) were present, telocytes formed a complex network establishing homocellular and heterocellular junctional complexes. The increase was relative to the biopsies of a non-dystrophic control and of a DMD patient, which displayed occasional interstitial telocytes. It is interesting to note that despite the number of ultrastructural studies on muscle biopsies of UCMD patients, an increase of telocytes has never been reported, suggesting a possible specific involvement at the interface of muscle with the epimysium/fascia. Telocytes were also increased in the matrix of the DF of the UCMD patient. Recently, telocytes have been reported in the human fascia lata [44]; however, their distribution and potential role in this tissue remain elusive. In other organs, telocytes contribute to tissue organization during development and maintain postnatal tissue/organ structural integrity and function [45]. Telocytes play an important role in cell–cell communication with adjacent cells within the tissue via direct patterns or through indirect ways, such as cellular junctions, the production of multi-factors, and extracellular vesicles [45,46]. In the context of skeletal muscle, an increase in telocytes has been reported in post-injury models in rodents, and particularly in exercise-induced remodeling after acute myocardial infarction [47]. In an ex vivo model of muscle injury, telocytes may activate and regulate satellite activity by invading the satellite cell niche with their telopodes through a fragmented basal lamina and contacting the underlying activated satellite cells [48]. Similar aspects were found in the biopsy of our UCMD patient. In fact, we found telopodes in direct contact with the muscle sarcolemma in areas with interrupted basement membrane.

These observations support the importance of these cells in differentiation and regenerative processes. However, it remains unclear why telocytes have never been reported/noted in other muscle-degenerative diseases characterized by active regeneration. We also did not notice an increase of these cells in the biopsy of the DMD patient, though DMD is a muscular disorder with regeneration as a prominent characteristic [49]. Telocytes were discovered recently, and their role with respect to specific pathomechanisms remains unknown. Telocytes may influence neighboring cells by secreting various signaling molecules [45]. Determination of whether the deficiency of collagen VI affects telocyte behavior/function requires further study.

## 5. Conclusions

The muscle biopsy/DF of the rectus femoris matches the peculiar pattern of outside–in muscle degeneration followed by fat substitution documented by muscle imaging. The changes at the interface of the muscle with the epimysium/DF are characterized by an early demise of the fast-twitch fibers and a marked ectopic fatty infiltration. One possible hypothesis is that peripheral muscle fibers, subjected to a more intense workload and a greater need for energy sources, are affected earlier by mitochondrial dysfunction associated with defective autophagy, the two pathogenetic mechanisms of collagen VI. In addition, the increase of telocytes in UCMD deep fascia may be the consequence of an active tissue-remodeling process, highlighting a possible unrecognized involvement/role of the DF in UCMD pathogenesis.

## Data Availability

Not applicable.

## Supplementary Materials

The following supporting information can be downloaded at: https://www.mdpi.com/article/10.3390/ijerph19031252/s1, Figure S1: A. Immunofluorescence analysis of collagen I on cross sections of muscle/deep fascia biopsy from a non-dystrophic control (CTRL), a Duchenne muscular dystrophy patient (DMD), and the UCMD patient (UCMD). In all samples, deep fascia (DF) appears as a fibrous sheet covering the muscle (M). Nuclei, DAPI. Scale bar, 50 m. B. Immunohistochemical analysis of the sarcolemmal component dystrophin (dys, green) and developmental myosin heavy chain (MHC-d, red) on muscle sections of the UCMD patient. Despite the presence of several hypotrophic fibers, only one show regeneration features (arrow). Nuclei, DAPI. Scale bar, 50 m. C. Immunohistochemical analysis of the basement membrane component nidogen (nidog, red) and neonatal myosin heavy chain (MHC-d, green) on muscle sections of the UCMD patient showing some small regenerating muscle cells. Nuclei, DAPI. Scale bar, 50 m. D. Ultrastructural analysis of UCMD muscle biopsy showing the presence of several telocytes (digitally colored in green) in the endomysium. A capillary vessel (Cv) appear almost completely wrapped by telocytes cytoplasmic processes (telopodes, Tp). Scale bar, 1 m.
