# Supplementary material for: Early Morphological Changes of the Rectus Femoris Muscle and Deep Fascia in Ullrich Congenital Muscular Dystrophy
Source: Int J Environ Res Public Health. 2022 Jan 23;19(3):1252. doi: 10.3390/ijerph19031252 (PMC8834967; doi:10.3390/ijerph19031252)
Supplement: Supplementary file 1 [file ijerph-19-01252-s001.zip › ijerph-1521593-supplementary.pdf]

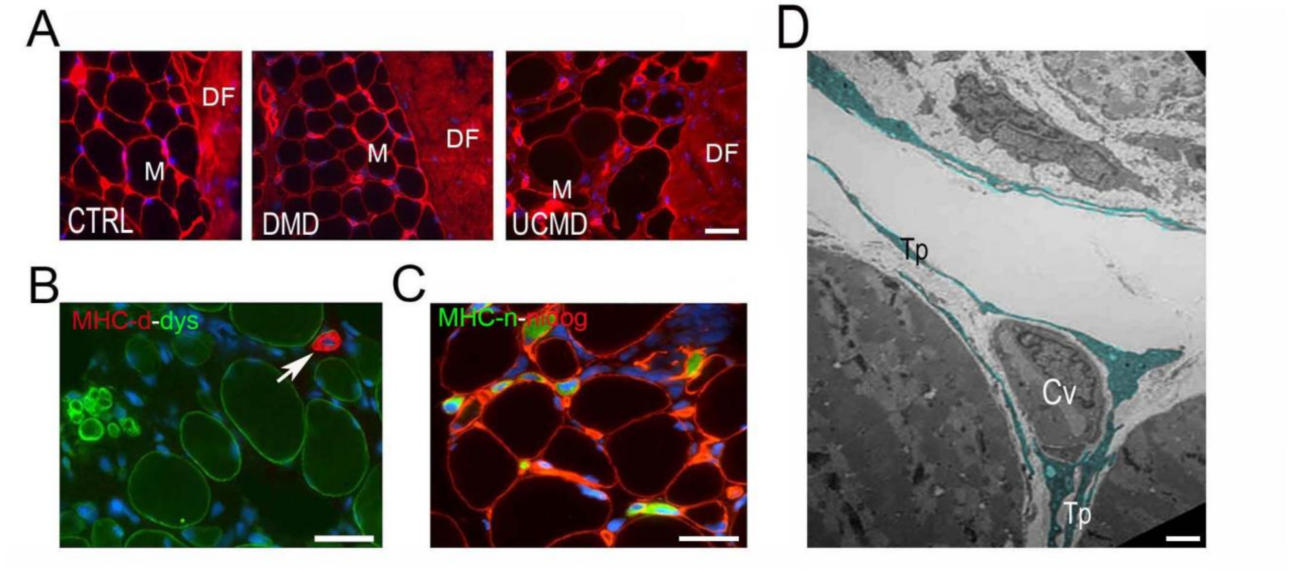

Figure S1. A. Immunofluorescence analysis of collagen I on cross sections of muscle/deep fascia biopsy from a non-dystrophic control (CTRL), a Duchenne muscular dystrophy patient (DMD), and the UCMD patient (UCMD). In all samples, deep fascia (DF) appears as a fibrous sheet covering the muscle (M). Nuclei, DAPI. Scale bar, 50 μm. **B**. Immunohistochemical analysis of the sarcolemmal component dystrophin (dys, green) and developmental myosin heavy chain (MHC-d, red) on muscle sections of the UCMD patient. Despite the presence of several hypotrophic fibers, only one show regeneration features (arrow). Nuclei, DAPI. Scale bar, 50 μm. **C.** Immunohistochemical analysis of the basement membrane component nidogen (nidog, red) and neonatal myosin heavy chain (MHC-d, green) on muscle sections of the UCMD patient showing some small regenerating muscle cells. Nuclei, DAPI. Scale bar, 50 μm. **D**. Ultrastructural analysis of UCMD muscle biopsy showing the presence of several telocytes (digitally colored in green) in the endomysium. A capillary vessel (Cv) appear almost completely wrapped by telocytes cytoplasmic processes (telopodes,Tp). Scale bar, 1 μm.
